# Implementation drivers scale: a new implementation measure to reduce mental health gaps

**DOI:** 10.1017/S146342362510025X

**Published:** 2025-07-15

**Authors:** Felipe Agudelo-Hernández, Marcela Guapacha-Montoya, Andrés Camilo Delgado-Reyes

**Affiliations:** 1 Ph.D in Social Sciences, Childhood and Youth, MD, Child and adolescent psychiatrist; Pan American Health Organization, Bogotá, Colombia; 2 M.D, Universidad de Caldas, Facultad de Ciencias para la Salud, Manizales, Caldas, Colombia; 3 Psychologist, PhD (c) in Psychology, Universidad de Manizales, Manizales, Colombia

**Keywords:** Community health planning, implementation science, mental health, primary health care, validation study

## Abstract

**Aim:**

The objectives of this study were to study the psychometric properties of the Implementation Drivers Scale (IDS), for the mhGAP programme, both clinical and community; to test its structural validity, and to propose an instrument to accompany the implementation of the mhGAP in similar contexts. For this purpose, a cross-sectional quantitative methodology study was conducted.

**Background:**

Mental health programmes proposed in low- and middle-income countries to address gaps in care have implementation problems.

**Methods:**

A cross-sectional quantitative methodology study was conducted. During 2022 and 2023, the instrument was administered to 204 individuals, including primary care professionals (50%), national administrative leaders (19.11%), and community strategy leaders. Three departments of Colombia participated, two with low levels of implementation in mental health programmes and one with high levels of implementation of programmes and services.

**Findings:**

The Kaiser-Meyer-Olkin factor analysis resulted in 0.861, which indicated the suitability of the data for a factor analysis. Bartlett’s Test of Sphericity had a value of 2480.907 (153 degrees of freedom, p <.001). The exploratory factor analysis explained variance of 66.781%. The four factors proposed in the AIF model (System enablers for implementation, Accessibility of the strategy, Adaptability and acceptability, and Strategy training and supervision) were confirmed, with all items with loadings greater than 0.4. For the entire instrument, a Cronbach’s alpha was 0.907. The IDS could contribute to the monitoring of some components of mhGAP implementation, both clinical and community-based, in low- and middle-income settings through appropriate validation processes.

## Introduction

Mental disorders and their complications show a progressive increase in low- and middle-income countries (Moitra *et al*., 2023) Despite this increase, the response continues to be insufficient, with few mental health professionals and few community-based programmes that promote recovery (Organización Panamericana de la Salud -OPS-, 2020).

In response to this problem, the World Health Organization has proposed the Mental Health Gap Global Action Program (mhGAP) (OPS, 2020), which includes a clinical and a community component, and seeks to strengthen the first level of mental health care and the community. as a recovery axis (Keynejad *et al*, 2021). When mhGAP is implemented properly, it presents good results in terms of recovery (Keynejad *et al.,* 2021).

However, despite having clearly defined components and an experience of approximately 15 years, the programme presents implementation problems in some Latin American contexts (Agudelo-Hernández and Rojas-Andrade, 2023; Sapag *et al*., 2021). These problems have been related to low resource allocation, limited training, low compliance, low supervision or support of health personnel, little involvement of administrative staff, low community participation, and cultural and demographic barriers (Agudelo-Hernández *et al.,* 2023; Beidas *et al.,* 2021)

To improve some of these implementation variables, some instruments have been proposed for community mental health programmes (Beidas *et al*., 2021) psychological first aid (Rojas-Andrade *et al.* 2023), primary health care programmes (Rojas-Andrade and Agudelo-Hernández, 2024), and even instruments to determine the involvement of decision-makers (Langlois *et al.,* 2016). Determining implementation is essential to identify barriers early, monitor the process to make adjustments to programmes, and contribute to a more accurate measurement of effectiveness (Proctor *et al.,* 2013). The above could serve to determine action plans that contribute early to better implementation (Michie *et al.,* 2009; Proctor *et al*., 2023). Thus, it is recommended that these implementation measures be validated, have easy application and interpretation, and can be integrated into health systems (Howard *et al.* 2017).

Several frameworks are proposed from implementation science to improve the feasibility, compliance, penetration and sustainability of programmes. These frameworks help overcome implementation problems and integrate evidence-based practices into health services (Howard *et al.* 2017). One of the most used frameworks is the Active Implementation Framework -AIF- (Clinton-McHarg *et al.,* 2016). This highlights aspects such as the participation of interested parties, training and support for executing professionals, permanent monitoring and feedback, leadership and organizational culture, and the fidelity of the components that constitute Evidence-Based Practices (Clinton-McHarg *et al.,* 2016).

This framework defines implementation stages that coincide with those proposed with mhGAP, including the exploration of the local context, the installation of the capacity and resources necessary to introduce and sustain the programme, the strategies for an initial implementation, and the implementation sustained with the highest possible quality (Bauer *et al.,* 2015; Fixsen *et al*., 2015). This also implies the allocation of resources and support from decision-makers (Clinton-McHarg *et al.,* 2016).

Multiple studies (Powell *et al.,* 2012; Walz *et al.,* 2019) have shown that this implementation framework could contribute to successful development of mental health programmes, which has led some researchers to use it to determine the effectiveness of Evidence-Based Practices and accompany their implementation (Beidas *et al.,* 2021). According to this implementation framework, the Implementation Drivers Scale (IDS) was designed, which includes from its theoretical development domains such as System enablers for implementation, Accessibility of the strategy, Adaptability and acceptability, and Strategy training and supervision.

Despite the existence of programmes to address the growing gap in treating mental disorders, there are implementation problems that need to be improved with measurement instruments. However, low- and middle-income countries, to which mhGAP is directed, lack validated instruments to plan and monitor the strategies that make up this programme (Beidas *et al.,* 2021; Langlois *et al.,* 2019). Some studies carried out in Latin America have shown that aspects such as training, continuous supervision and management by administrative staff are essential to address for an adequate implementation of mhGAP (Agudelo-Hernández *et al.,* 2024).

These studies have generated measurement instruments that determine the components of the mental health strategy implementation process, including contextual factors, training protocols, implementation climate, and long-term sustainability. These instruments are based on implementation frameworks such as the Active Implementation Framework (AIF) (Rojas-Andrade & Agudelo-Hernández, 2024). These instruments have demonstrated adequate concurrent validity with other health quality measures, such as the OR4KT (Shea *et al*., 2014), adequate reliability (Cronbach’s alpha of 0.914), and adequate construct validity, with a Fleiss’s kappa index of 0.81–1.00, which indicates adequate agreement in the consensus among experts (Rojas-Andrade and Agudelo-Hernández, 2024). This allowed for the development of the instrument consisting of 18 items, presented on a five-point Likert scale.

### Research objective

The objectives of the research were: 1. To study the psychometric properties of the IDS, for the mhGAP programme, both clinical and community 2. To test its structural validity, and 3. To propose an instrument to accompany the implementation of the mhGAP in similar contexts.

## Method

A cross-sectional quantitative methodology study was conducted, that aimed to evaluate the capacity of the IDS scale to determine components of mhGAP implementation.

### Participants and Procedure

The participants were 204 people (50% were men and 50% women). 102 were health personnel trained in the mhGAP programme (clinical component of mhGAP), 39 were territorial mental health referents (person who leads the administration of the mental health plans of the departments of Colombia, which are 32 in total) and 63 were leaders of support groups, some belonging to community mental health platforms (community component of mhGAP).

78.43% of the participants registered a health profession. Three departments of Colombia participated. Two with low levels of implementation in mental health programmes (La Guajira and Chocó) and one with high levels of implementation of programmes and services (Caldas) (Agudelo-Hernández and Rojas-Andrade, 2023). These departments were chosen because they have a different level of implementation and because they recently started the mhGAP.

The sample was a convenience, according to the possibility given by the mhGAP training led by the Ministry of Health and Social Protection and Pan American Health Organization. This application was carried out in more than 80% of the training courses carried out in the country at the time of the study.

The application was carried out in a standard way during the mhGAP training, as part of the posttest proposed by the programme, in the years 2023 and 2024. Although people could refuse to answer the instrument, without this compromising their certification in the programme, they all answered the instrument. The mhGAP criteria for training were considered as eligibility criteria. Thus, the instrument was directed to physicians, nurses, social workers and psychologists, at the first level of health care, in the case of the clinical component. To social leaders in mental health, in the case of the community component, and to administrative leaders in the programme management component. At all times, confidentiality was respected and the data were only handled by the research team, the Ministry of Health and the Pan American Health Organization, Colombia.

The instrument was applied by programme trainers, belonging to the Ministry of Health of Colombia and the Pan American Health Organization of Colombia. Participation was voluntary and was applied only if the person agreed to be part of the study. All participants completed the entire scale and there were no missing data.

### Instrument

The instrument consists of a scale with 18 items, which is answered with a Likert scale (0 = Strongly disagree, 1 = Disagree, 2 = Neither agree nor disagree, 3 = Agree, 4 = Strongly agree). Each item investigates the application, according to each role, of the mhGAP implementation components. This instrument can be completed by health personnel at the clinical and administrative levels and by community mental health leaders (Appendix I).

This scale consists of four domains, called *System enablers for implementation* (F1)*, Accessibility of the strategy* (F2)*, Adaptability and acceptability* (F3), and *Strategy training and supervision* (F4). The four domains have a Cronbach’s alpha greater than 0.725, in addition to a McDonald’s Omega of 0.904, 0.863, 0.714 and 0.808, respectively (Rojas-Andrade and Agudelo-Hernández, 2024).

These domains were based on and coincide with the Active Implementation Framework (AIF), which proposes exploration, installation, initial Implementation and full Implementation as moments of adequate implementation (Fixsen *et al.,* 2015). This implementation framework has shown effectiveness in improving the quality of life and the functioning of mental health services (Fixsen *et al.,* 2015; Proctor *et al*., 2013)

Cognitive interviews were conducted as a semi-structured interview to obtain information about the difficulties that the interviewee has faced in relation to understanding the questions, retrieving information and elaborating responses. These questions were asked to one person from each profession, in the case of clinical mhGAP, and to one person from the community component and with an administrative role. The methodology was developed with verbalization of thought, tests focused on meaning, paraphrasing, evaluation of questions and classification of vignettes (Caicedo-Cavagnis and Zalazar-Jaime, 2018).

### Analysis

The SEM (Structural Equations Modeling) analysis of the IDS was carried out with the items as units of analysis to interpret the variance of each construct. Due to having categorical variables and with various degrees of asymmetry and kurtosis, a polychoric correlation matrix was created between these items (Lei and Wu, 2012) for the identification of the model. The Maximum Likehood estimator was also applied and the Satorra-Bentler procedure (SB-χ2) to adapt the goodness-of-fit test because they are abnormal variables (Lloret-Segura *et al*., 2014)

To assess the fit, a confirmatory phase was carried out that was based on the hypothesis of the structure of the AIF model, in its Implementation Drivers component. This structure is composed of the domains of development of relevant competencies, necessary organizational supports, and engaged leadership. Subsequently, another exploratory phase, which allowed the use of several indicators (Hu and Bentler, 1999): in addition to the χ2 goodness-of-fit test, such as absolute fit indices (root mean square error Gof approximation, RMSEA ≤ 0.05; standardized mean residual, SRMR ≤ 0.08) and incremental fit (Comparative Fit Index, CFI ≥ 0.95; Tucker Lewis Index, TLI ≥ 0.95).

In this sense, several measurement models were tested: Single factor model, four oblique factor model and a hierarchical model, based on a theoretical justification of the AIF model, in which all factors contribute to promote the implementation of a health programme. Reliability was estimated with Cronbach’s alpha. The statistical programme SPSS (version 26 and AMOS) was used.

### Ethical considerations

This work complies with the standards for research in human beings as provided in resolution No 008430 of 1993 of the Ministry of Health and in the Declaration of Helsinki of 2000. It is a minimal-risk investigation and was reviewed and endorsed by document CBE02_2022 by the Bioethics Committee of the [masked].

## Results

The Content validity index (CVI) for each item was based on the formula CVI = (ne − N/2)/N/2, where *ne* is the number of evaluations of the item as essential and N is the total number of evaluations. The CVI ranges between +1 and −1, with positive scores indicating better content validity. All the items met the parameters of clarity, objectivity, relevance, organization, sufficiency, adequacy, consistency, coherence, methodology, and significance, with an Aiken V between .90 and .99 for all the questions in the instrument.

In the cognitive test, understanding of the question was found in all people to whom the instrument was applied, with no differences according to profession or role. There were no differences between what was understood by community leaders, clinical health professionals and administrative professionals. A similar paraphrasing was found, all eight people explained it and no difficulties were reported in understanding any question.

Answers were found with all the options for each question. The average values of the factors were between three and five. The kurtosis of the factors was also similar, with platykurtic distributions. According to the symmetry in the distribution, all items showed a positive asymmetry (between 2.47 and 3.25) (Table I).

**Table I.** Descriptive statistics and polychoric correlations for the items

Item	1	2	3	4	5	6	7	8	9	10	11	12	13	14	15	16	17	18
1	1	0,62	0,626	0,349	0,511	0,453	0,474	0,336	0,333	0,289	0,379	0,28	0,323	0,302	0,365	0,07	0,233	0,226
2		1	0,681	0,495	0,712	0,672	0,597	0,313	0,209	0,085	0,265	0,125	0,086	0,201	0,263	−0,06	0,181	0,176
3			1	0,306	0,587	0,528	0,528	0,413	0,418	0,325	0,475	0,355	0,299	0,37	0,377	0,01	0,214	0,16
4				1	0,442	0,546	0,438	0,189	0,097	0,011	0,222	0,129	0,028	0,157	0,201	0,19	0,273	0,245
5					1	0,688	0,494	0,231	0,269	0,089	0,386	0,234	0,07	0,243	0,312	0,01	0,269	0,129
6						1	0,72	0,304	0,21	0,039	0,349	0,201	0,009	0,194	0,22	−0,05	0,155	0,097
7							1	0,405	0,304	0,167	0,349	0,255	0,266	0,3	0,306	−0,05	0,139	0,021
8								1	0,733	0,522	0,5	0,474	0,466	0,42	0,385	0,05	0,116	0,209
9									1	0,651	0,589	0,58	0,596	0,539	0,491	0,02	0,153	0,16
10										1	0,618	0,68	0,681	0,645	0,491	0,18	0,182	0,239
11											1	0,779	0,56	0,647	0,58	0,23	0,301	0,23
12												1	0,738	0,775	0,562	0,28	0,298	0,304
13													1	0,81	0,609	0,38	0,281	0,283
14														1	0,622	0,33	0,416	0,325
15															1	0,36	0,411	0,38
16																1	0,468	0,371
17																	1	0,569
18																		1
M	3,11	3,24	3,01	3,22	3,19	3,25	3,08	2,95	2,89	2,7	2,58	2,47	2,64	2,53	2,7	3,13	2,9	2,92
DE	1,023	1,005	1,083	0,855	0,919	0,962	1,016	1,135	1,178	1,198	1,1	1,142	1,242	1,193	1,094	0,784	0,896	0,999
As	−0,842	−1,085	−0,702	−0,955	−0,927	−1,113	−0,851	−0,842	−0,812	−0,367	−0,102	0,015	−0,123	−0,038	−0,433	−0,476	−0,17	0,846
Ku	−0,188	0,163	−0,679	0,973	0,279	0,317	−0,198	−0,276	−0,31	−0,813	−1,139	−1,057	−1,108	−1,1	−0,515	−0,526	−0,107	5,978

As = Asymmetry; Ku = Kurtosis. The authors.

The Kaiser-Meyer-Olkin factor analysis resulted in 0.861, which indicated the suitability of the data for a factor analysis. Bartlett’s Test of Sphericity had a value of 2480.907 (153 degrees of freedom, p <.001). The exploratory factor analysis was performed with extraction of principal and rotated components, with an explained variance of 66.781%. The four factors proposed in the AIF model were confirmed, with all items with loadings greater than 0.4. For the entire instrument, a Cronbach’s alpha was 0.907, acceptable for the four domains proposed by the instrument, as shown in Table II.

**Table II.** Rotation of IDS factors. Communalities, explained variance and Cronbach’s Alpha total and of each factor

*Item*	*F1*	*F2*	*F3*	*F4*	*Communalities*
1	0,662				0,534
2	0,798				0,784
3	0,893				0,645
4	0,759				0,527
5	0,706				0,682
6.	0,773				0,75
7		0,7			0,628
8		0,673			0,569
9		0,698			0,721
10		0,682			0,731
11		0,671			0,679
12		0,601			0,759
13			0,693		0,781
14			0,678		0,753
15			0,899		0,604
16				0,571	0,624
17				0,761	0,687
18				0,705	0,563
*Cronbach’s alpha*	.917	.898	.609	.716	
*McDonald’s Omega*	.899	.867	.702	.727	
*Number of items*	6	6	3	3	
*Explained variance*	39.274%	17.400%	5.054%	5.053%	
*Explained variance total*					66.781%
*Cronbach’s Alpha total*					.907
*Kaiser-Meyer-Olkin*					.861

The authors.

The item-total and item-total correlation matrix is provided in Supplement 1, which allows us to see the magnitude of the correlation and the direction in which it is. This item-total correlation expresses a favourable relationship across the scale, providing a degree of coherence when assessing the proposed domains, which made it possible to construct an instrument with a proposed multidimensional structure. Regarding the models, in the four oblique factors model, a correlation was found between the four factors, which speaks of the convergence between them. The discrimination indices of the items were lower between F1 and F3 (r = 0.948, p < 0.01). The hierarchical model showed better results, with RMSEA less than 0.08, indicating that the IDS factors should be interpreted jointly (Figure I).

**Figure 1.**
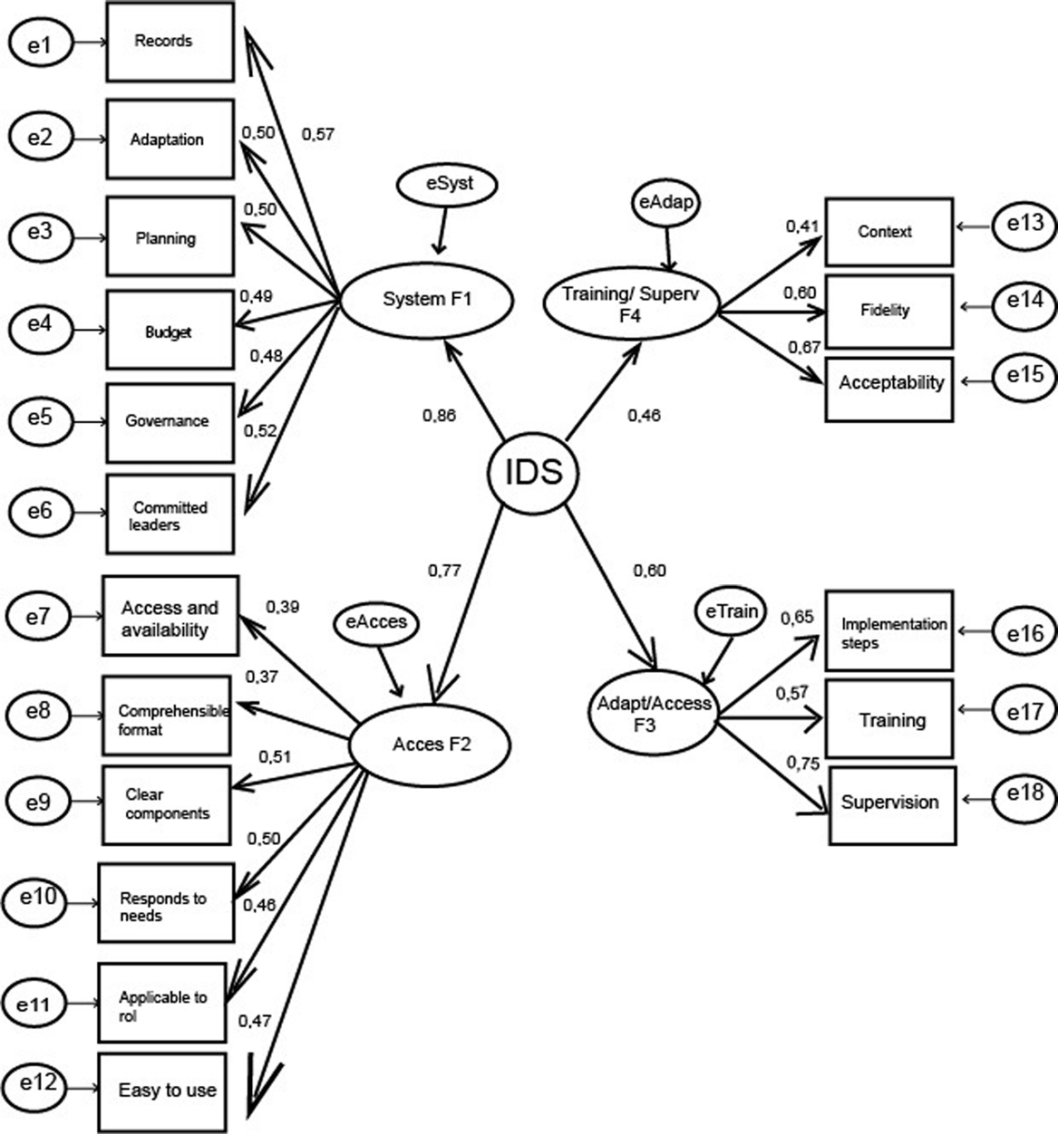
Estimates of the model parameters. SB-χ2 (gl) = *279.820; RMSEA =* 0,059; *CFI =* 0,869. Estimates of the Model parameters. The authors.

## Discussion

The present research managed to describe the psychometric properties and identify the factor structure of the IDS in a sample of mental health professionals, administrative coordinators and leaders of community mental health devices, in Colombian territories with different levels implementation of mental health programmes. The results indicate IDS could be used in the planning and monitoring of mhGAP.

Regarding the hypothesis that the study wanted to test, initially proposed by Rojas-Andrade and Agudelo-Hernández (2024), the structure of the scale found in the present study confirms the hypothesis with which the model was built, considering the components of the AIF implementation framework: development of competencies, necessary organizational supports, and engaged leadership.

This is supported by the high reliability and by factor loadings greater than 0.5 in all items, in addition to the contribution of all items to their respective domains. The interpretation of the instrument can be done with a hierarchical model, which considers all the implementation variables of the AIF framework, since when using the domains independently, poor discriminant validity is found (Farrell, 2010).

By considering all factors, this instrument aims to highlight frequent barriers to mhGAP implementation, to improve management, training and supervision. This is essential, since it has been determined that, although a hundred countries have been using the programme, it has been carried out mainly in training, and not in the monitoring or management stage (Agudelo-Hernández *et al.,* 2023; Keynejad *et al.,* 2021).

To which are added other factors that influence implementation, such as cultural barriers, armed conflict, migration (Agudelo-Hernández *et al.,* 2021; Ghasemi *et al.,* 2021), in addition to few resources assigned to mental health and the majority of mental health resources assigned to the provision of hospital services, with low implementation of primary mental health care and community health (OPS, 2020; Waltz *et al.,* 2019). Likewise, this instrument points to implementation challenges, such as the adaptations of the components to the contexts without losing compliance, which, added to acceptability (Murphy *et al.,* 2022) is significantly linked to the results of a strategy (Aarons *et al.,* 2011; Michie *et al.,* 2009).

The IDS is proposed as an instrument that can be understood by different roles and with various educational levels, which could facilitate monitoring of community mental health strategies and from the first levels of health care. Other instruments have been proposed to determine the implementation of mental health programmes (Grandes *et al*., 2017; Rojas-Andrade *et al.*, 2023; Waltz *et al*. 2019). Although there is no scale to assess these components of mhGAP implementation, there are other comparable instruments that have been used to assess community mental health interventions, and one of their components is the performance of professionals. These include Therapy Procedures Observational Coding System – Strategies and Enhancing Assessment of Common Therapeutic factors (Kohrt *et al*., 2015).

Furthermore, in this validation of the instrument, a Cronbach’s alpha higher than that of the initial instrument was found (0.907 vs 0.892) (Rojas-Andrade and Agudelo-Hernández, 2024). However, these instruments are designed to evaluate the performance of professionals, which could escape the community mhGAP, which is a key component of the programme, and is aimed at community leaders.

The present study proposes a tool to facilitate the development of primary health care and community care, both priority aspects in the actions to be carried out in terms of mental health (Moitra *et al*., 2023; OPS, 2020; Patel *et al*., 2018). Carrying out these objectives requires considering the intersection of various determinants of quality of life and well-being, with the adequate management of health services (Ghasemi *et al*., 2021).

Although the regions where the study was carried out have different levels of implementation of mental health services (Agudelo-Hernández and Rojas-Andrade, 2023), another limitation could be considered the sample restricted to three territories, as it might not address implementation challenges such as interculturality and geographic diversity. Another limitation could be considered the sample. Although the recommended limit for the factor analysis of the items is 200 (Murphy *et al.,* 2022) it shows that it exceeds the present study, it could be carried out in other regions so that the results are more general at the level of a country. Likewise, we point out as a limitation the use of a convenience sample, which, although representative of several regions in Colombia, may generate selection bias.

Likewise, caution regarding the generalizability of results would be necessary without adaptation and validation processes to other low- and middle-income contexts, for which the mhGAP is proposed. Future studies could also consider simulating possible scenarios, as scenario analysis can inform the development and validation of implementation tools. By simulating different conditions and their potential impacts on mental health care delivery, it may be possible to refine instruments to better address real-world challenges faced by professionals, especially in contexts with cultural variations and with different forms of implementation of mental health services.

The implications of this study are that it could contribute to addressing an implementation problem by measuring key aspects such as the competencies of the first level of care teams, necessary organizational supports, and engaged leadership. These components are recognized as drivers of mhGAP implementation and have few simple monitoring and follow-up mechanisms.

Future studies, apart from considering adaptation to other contexts, could address an important limitation of the present research, which is recognized by the absence of a more forceful participation of people with mental disorders in the design of tools to monitor mhGAP in its clinical and community components. Although this study addressed a community component, where people with lived experience and leaders of peer groups participated, they were not the majority. Future studies should address the psychometric variables addressed in this study in other contexts where the mhGAP is used.

In conclusion, the present study shown that the theoretical model proposed for IDS is adequate for this context, which is given by the conformation of the factors based on the scale variables. These variables have been proposed from an implementation framework (AIF) that facilitates planning, training, appropriation and sustainability of the mhGAP, in addition to the acceptability of the programme by people with mental disorders and by health professionals and managers themselves. It was also found that these variables have statistically significant loadings.

The facilitators for the implementation of mhGAP, a WHO programme to reduce mental health gaps in low- and middle-income countries, could be given by System enablers for implementation, Accessibility of the strategy, Adaptability and acceptability, and Strategy training and supervision. This instrument, with adaptation and validation to diverse contexts, could favour the adequate and timely implementation of these components.

## Supporting information

Agudelo-Hernández et al. supplementary material 1 Agudelo-Hernández et al. supplementary material

Agudelo-Hernández et al. supplementary material 2 Agudelo-Hernández et al. supplementary material

## Supplementary material

The supplementary material for this article can be found at https://doi.org/10.1017/S146342362510025X
