# Supplementary material for: Implementation drivers scale: a new implementation measure to reduce mental health gaps
Source: Prim Health Care Res Dev. 2025 Jul 15;26:e57. doi: 10.1017/S146342362510025X (PMC12281048; doi:10.1017/S146342362510025X)
Supplement: Agudelo-Hernández et al. supplementary material 1 — Agudelo-Hernández et al. supplementary material [file S146342362510025Xsup001.docx]

*Appendix 1. IDS in its original version.*

Instrucción: Marque con una X la opción más adecuada según su experiencia en la aplicación del mhGAP. 0=Muy en desacuerdo, 1=En desacuerdo, 2=Ni de acuerdo ni en desacuerdo, 3=De acuerdo, 4=Muy de acuerdo

| Ítem | 0 | 1 | 2 | 3 | 4 |
| --- | --- | --- | --- | --- | --- |
| Es de fácil acceso y está siempre disponible para su uso. |  |  |  |  |  |
| Tiene un formato atractivo y fácil de comprender. |  |  |  |  |  |
| Expresa de manera clara, precisa e inequívoca los componentes o acciones inmodificables de las intervenciones. |  |  |  |  |  |
| Conozco en detalle todas y cada uno de los pasos para realizar la estrategia en mi territorio |  |  |  |  |  |
| Cumple mis expectativas y ayuda a responder a mis necesidades técnicas. |  |  |  |  |  |
| Es aplicable a mi rol como referente o apoyo al referente |  |  |  |  |  |
| Es fácil de usar e implementar |  |  |  |  |  |
| He recibido la capacitación suficiente para aprender a aplicarla de manera correcta y completa |  |  |  |  |  |
| He recibido supervisión y acompañamiento suficiente para aplicar de manera correcta y completa |  |  |  |  |  |
| Los registros estadísticos y sistemas informáticos que utilizo están alineados con mhGAP |  |  |  |  |  |
| La planificación anual de mi equipo considera la estrategia mhGAP |  |  |  |  |  |
| El presupuesto anual de mi equipo se define tomando en cuenta mhGAP |  |  |  |  |  |
| Otras autoridades locales de salud favorecen la implementación de la mhGAP |  |  |  |  |  |
| Mis jefes directos están comprometidos con la implementación de la estrategia mhGAP |  |  |  |  |  |
| Adecúo mhGAP a las características de mi contexto sin modificar los componentes esenciales |  |  |  |  |  |
| Cambio los componentes esenciales de mhGAP para adaptarlos a mi contexto |  |  |  |  |  |
| Aplico el ABC de mhGAP de manera correcta y completa |  |  |  |  |  |
| Las personas usuarias están satisfechas y reciben bien la atención basada en mhGAP |  |  |  |  |  |

The authors.
