# Supplementary material for: Implementation drivers scale: a new implementation measure to reduce mental health gaps
Source: Prim Health Care Res Dev. 2025 Jul 15;26:e57. doi: 10.1017/S146342362510025X (PMC12281048; doi:10.1017/S146342362510025X)
Supplement: Agudelo-Hernández et al. supplementary material 2 — Agudelo-Hernández et al. supplementary material [file S146342362510025Xsup002.docx]

**SUPPLEMENTARY MATERIALS**

**Appendix 1. Summary of control variables**

In this appendix we describe the macro- and micro-level control variables used in our analyses, as well as the expectations for the effects of these variables. A summary of variable measurement is found in Appendix Table 1.1, and descriptive statistics for all variables are reported in Appendix Table 1.2.

**Control variables: Macro-level variables**

***Immigrant population***. We include as an independent variable in our models the percentage of the foreign-born population in the total state population. Data on the foreign-born population are collected from the Current Population Survey (CPS). We expect this variable to have a negative effect on support for welfare spending.

***Black population***. The percentage of African American population is included as a macro-level control variable in order to explore whether or not the presence of African Americans influences public support for welfare spending. Data on the Black population percentage are collected from the Current Population

Survey (CPS). Following previous research, we suggest that this variable should be negatively associated with support for welfare spending.

***Blacks on welfare***. In order to capture the reality of Blacks’ welfare participation at the state level, we use data from the CPS-ASES March Survey to tabulate the percentage of Black households participating in any major welfare programs by state and year.^^[[1]](#endnote-1)^^ We expect the coefficient for this variable to be negative.

***Real GDP per capita, unemployment rate, and state government ideology***. Cutright ([1965](#_ENREF_10)), Wilensky ([1975](#_ENREF_30)), and Quadagno ([1994](#_ENREF_26), [2003](#_ENREF_8)) contend that macroeconomic conditions influence individuals’ attitudes toward government spending. Hence we include both real per capita income (in 2005 dollars) and the unemployment rate as macro-level control variables in the model. Since real per capita income is a measure of state wealth, we expect the coefficient for this variable to be negative, indicating that individuals from wealthy states are less likely to support greater welfare spending. On the other hand, we hypothesize that the state unemployment rate will be positively related to support for welfare spending, since unemployment may be an indicator of state need for welfare programs. Data on real per capital income and the unemployment rate are collected from the U.S. Census Bureau. Finally, we speculate that individuals residing in states with a more liberal state government will support higher levels of government spending in general and, more specifically, in support of the poor; hence we also include state government liberalism (Berry, Ringquist, Fording, and Hanson [1998](#_ENREF_7)) as a control variable in our models.

***Control Variables: Micro-level variables***

***Attitudes toward Blacks and the poor***. Gilens (1999) demonstrates that attitudes toward welfare spending are influenced by individuals’ attitudes toward Blacks, so a feeling thermometer variable for Blacks is included as a statistical control; this variable ranges from 0 (negative views toward Blacks) to 100 (positive views toward Blacks). Since welfare programs are designed for low-income individuals, we also include in our model a control variable for attitudes toward the poor, measured using the traditional feeling thermometer scale ranging from 0 (negative views toward the poor) to 100 (positive views toward the poor). The coefficients for each of these variables should be positive, indicating that individuals who hold favorable attitudes toward Blacks and the poor should be more supportive of welfare spending.

***Political ideology and partisanship.*** Symbolic politics variables have also been found to affect individuals’ support for government spending ([Jacoby, 1994](#_ENREF_19); [Henderson, Monroe, Garand, and Burts,](#_ENREF_16)

[1995](#_ENREF_16); [Bean and Papadakis, 1998](#_ENREF_6); [Edlund, 1999](#_ENREF_11); [Jacoby, 2000](#_ENREF_18); [Pettersen, 2001](#_ENREF_25)). To account for these effects, we include two variables in our models: (1) a seven-point partisan identification scale, ranging from 0 (strong Democrat) to 6 (strong Republican); and (2) political ideology, measured on a seven-point scale ranging from 0 (strong liberal) to 6 (strong conservative). We hypothesize that the coefficients for these two variables will be negative, suggesting that Republicans and conservatives will be the less favorable in their attitudes toward welfare.

***Demographic attributes***. Lowery and Berry ([1983](#_ENREF_22), [2009](#_ENREF_5)), Sihvo and Uusitalo ([1995](#_ENREF_27)), and Lewis-Beck and Rice ([1985](#_ENREF_21)) argue that, out of their own self-interest, groups with a relatively high share of consumers of welfare (such as the elderly, unemployed, minorities, children, women, low-income and/or poorly-educated individuals) support government spending on welfare more than others. Moreover, van Oorschot ([2006](#_ENREF_29)) contends that religion plays a role in solidarity toward the poor, thus suggesting that individuals who attend church services more often are more likely to support programs for needy people. Given this body of previous research, we include in our models a series of demographic control variables, including separate variables for Black, Hispanic, and Asian respondents, age, gender, education, family income, and church attendance. Given the findings of previous research, we suggest that Blacks, Hispanics, Asians, women, the elderly, and individuals with high levels of church attendance will be more supportive of welfare spending than other individuals, while high-income individuals and the highly-educated will be less favorably oriented toward welfare.

**Appendix Table 1.1. Description of Variables**

______________________________________________________________________________________­­­­

Variable Description

______________________________________________________________________________________

Support for welfare spending 2 = respondent supports increases in welfare spending; 1 = respondent supports keeping welfare spending the same; 0 = respondent supports decreases in welfare spending.

Percent immigrants on welfare Number of immigrant households on welfare as a percentage of total number of immigrant households (Source: Current Population Survey Annual Social and Economic Supplements [CPS-ASES]). We identify immigrant households that used one or more of the following welfare programs in the past 12 months: (1) food assistance programs such as food stamps, reduced and free lunch, and WIC assistance; (2) public assistance cash benefits; (3) housing assistance programs such as public housing or renting subsidy; and (4) Medicaid.

Percent immigrants on welfare (alternative) Number of immigrant households on welfare as a percentage of total number of welfare households (Source: Current Population Survey Annual Social and Economic Supplements [CPS-ASES]). We identify immigrant households that used one or more of the following welfare programs in the past 12 months: (1) food assistance programs such as food stamps, reduced and free lunch, and WIC assistance; (2) public assistance cash benefits; (3) housing assistance programs such as public housing or renting subsidy; and (4) Medicaid.

Support for immigration Support for increased immigration: 4 = respondent strongly supports increased immigration; . . . ; 0 = respondent strongly supports decreased immigration.

Feeling thermometer: illegal immigrants Feeling thermometer for illegal immigrants: 100 = strong positive affect; . . .; 0 = strong negative affect.

Pro-immigration scale Factor score for principle components analysis of support for immigration and feeling thermometer for illegal immigrants (Eigenvalue = 1.339, variance explained = 0.670).

Percent immigrants Foreign-born population as a percentage of total state population (Source: Current Population Survey [CPS]).

Percent Blacks African Americans as a percentage of total state population (Source: U.S. Census Bureau Statistics Abstract).

**Appendix Table 1.1 (continued)**

______________________________________________________________________________________­­­­

Variable Description

______________________________________________________________________________________

Percent Blacks on welfare Number of Black households on welfare as a percentage of total number of Black households (Source: CPS-ASES).

Unemployment rate Percentage of state population that is unemployed (U.S. Census Bureau Statistics Abstract).

Real per capita income Deflated per capita income (Source: Bureau of Economic Analysis).

State government ideology Collective ideological orientation of state legislators and governors (Source: Berry et al. 1996).

Feeling thermometer: Blacks Feeling thermometer for Blacks or African Americans: 100 = strong positive affect; . . .; 0 = strong negative affect.

Feeling thermometer: the poor Feeling thermometer for the poor: 100 = strong positive affect; . . .; 0 = strong negative affect.

Partisan identification 7-point partisan identification scale: 6 = respondent is a strong Republican; . . . ; 0 = respondent is a strong Democrat.

Political ideology 7-point scale of liberal-conservative ideology: 6 = respondent is a strong conservative; . . . ; 0 = respondent is a strong liberal.

Gender 1 = respondent is a female; 0 = respondent is a male.

Black respondent 1 = respondent is Black or African American; 0 = otherwise.

Hispanic respondent 1 = respondent is Hispanic or Latino; 0 =otherwise.

Asian respondent 1 = respondent is Asian; 0 = otherwise.

Education 7 = respondent has post-graduate degree; . . . ; 1 = respondent has less than a high school degree

Family income 5-point family income scale: 5 = respondent is in top 5% of income distribution; . . . ; 1 = respondent is in bottom one-sixth of income distribution.

**Appendix Table 1.1 (continued)**

______________________________________________________________________________________­­­­

Variable Description

______________________________________________________________________________________

Age Respondent age (in years)

Church attendance 4 = respondent attends church services more than once a week; . . . ; 0 = respondent never attends church services.

**Appendix Table 1.2: Descriptive Statistics of Key Variables**

Variables N Mean Std. Dev. Min Max

**____________________________________________________________________________________________________________**

Support for welfare spending 9,378 0.741 0.734 0 2

Immigration attitudes 9,378 0.088 1.014 -1.71 2.71

Support for immigration 9,378 1.554 1.087 0 4

Feeling thermometer: illegal immigrants 9,378 41.246 26.602 0 97

Percent foreign-born 9,378 12.553 7.629 1.25 27.30

Percent foreign-born on welfare 9,378 42.522 6.863 19.44 60

Percent Blacks 9,378 12.378 8.867 0.22 44.08

Percent Blacks on welfare 9,378 54.888 7.847 20 83.33

Unemployment rate 9,378 6.622 1.910 2.8 11.2

Real per capita income 9,378 19419.940 2789.557 14191.11 28878.74

State government ideology 9,378 43.173 18.293 17.51 73.13

Feeling thermometer: Blacks 9,378 67.868 20.642 0 97

Feeling thermometer: the poor 9,378 70.403 19.211 0 97

Partisan identification 9,378 2.745 2.163 0 6

Political ideology 9,378 3.171 1.502 0 6

Gender 9,378 0.504 0.500 0 1

Race: Black 9,378 0.126 0.332 0 1

Ethnicity: Hispanic 9,378 0.139 0.346 0 1

Race: Asian 9,378 0.022 0.146 0 1

Age 9,378 48.708 17.420 0 93

Education 9,378 3.640 1.392 0 5

Family income 9,378 1.931 1.124 0 4

Church attendance 9,378 1.609 1.602 0 4

**____________________________________________________________________________________________________________**

**Appendix Table 2. Multi-level ordered logit model estimates for support for public welfare in the United States, 2004-2016, without control variables**

**_____________________________________________________________________________________________________________________**

Without state fixed effects With state fixed effects

------------------------------------------------------- -------------------------------------------------------

Model 1 Model 2 Model 3 Model 4

------------------------ ----------------------- ------------------------ -----------------------

b z b z b z b z

**_____________________________________________________________________________________________________________________**

Immigration attitudes 2.936 30.91*** 1.560 3.64*** 2.929 30.76*** 1.530 3.57***

Percent foreign born on welfare -0.758 -3.04*** -1.634 -4.49*** -1.312 -3.68*** -2.201 -4.96***

Percent foreign born on welfare * --- --- 2.149 3.29*** --- --- 2.186 3.33***

immigration attitudes

**_________________________________________________________________________________________________________________________________**

N  9,378 9,378 9,378 9,378

Wald χ2 960.61 967.11 996.11 1002.90

Prob (Wald χ2) 0.0000 0.0000 0.0000 0.0000

Log likelihood -9102.863 -9097.467 -9083.472 -9077.921

*** prob < 0.001 ** prob < 0.01 * prob < 0.05

**Appendix Table 3. Multi-level ordered logit model estimates (with fixed effects) for support for public welfare spending in the United States, 2004-2016, with alternative measure of immigrant welfare participation.**

____________________________________________________________________________________

Model 1 Model 2

-------------------------- --------------------------

b z b z

____________________________________________________________________________________

Immigration attitudes 1.846 16.74*** 1.376 7.33***

Percent foreign born on welfare -3.209 -3.01*** -3.693 -3.45***

Percent foreign born on welfare *

immigration attitudes --- --- 1.072 3.07***

Percent foreign born 3.138 1.83* 3.164 1.86*

Percent blacks -0.009 -0.23 -0.010 -0.26

Percent blacks on welfare 0.397 0.22 0.482 0.27

Unemployment rate -0.130 -6.42*** -0.129 -6.46***

Real per capita income -0.0002 -4.91*** -0.0002 -4.95***

State government ideology 0.016 5.19*** 0.015 5.17***

Feeling thermometer: blacks 0.0004 0.35 0.0003 0.26

Feeling thermometer: the poor 0.011 8.83*** 0.011 8.75***

Partisan identification -0.180 -13.21*** -0.179 -13.12***

Political ideology -0.304 -15.74*** -0.304 -15.71***

Gender 0.112 2.62** 0.114 2.66**

Race: black 0.456 6.20*** 0.469 6.37***

Ethnicity: Hispanic -0.005 -0.06 -0.022 -0.31

Race: Asian 0.259 1.76* 0.269 1.83*

Age -0.001 -1.03 -0.001 -0.93

Education -0.095 -5.74*** -0.093 -5.56***

Family income -0.257 -12.58*** -0.258 -12.61***

Church attendance 0.010 0.70 0.009 0.63

____________________________________________________________________________________

N 9,378 9,378

Wald χ^2^ 2426.72 2435.54

Prob (Wald χ^2^) 0.0000 0.0000

Log likelihood -8148.012 -8143.27

*** prob < 0.001 ** prob < 0.01 * prob < 0.05

Note: The model upon which these estimates are based includes (1) an alternative measure of immigrant welfare participation based on the number of immigrants on welfare as a share of all state welfare recipients, and (2) state-level fixed effects.

**Appendix 4: Using IV-2SLS to Address Endogeneity and Reciprocal Causality Issues**

Even though our empirical analyses show strong support for our hypotheses, we find it imperative to address the problem of reverse causality, or endogeneity, between immigration attitudes and welfare support. Especially, predicting welfare support based on other attitudes is inherently problematic in terms of determining the direction of causality (Fordham and Kleinberg 2012). In our case, it is possible that individuals’ welfare attitudes are influenced by their immigration attitudes; however, it is also possible that individuals choose to dislike immigrants because of welfare-related concerns. In order to better test our hypothesis, we need to ensure the effect of immigration attitudes on welfare support is robust even when accounting for the possibility of reverse causality. To do this, we employ a two-stage least-squares model with instrumental variables (IV-2SLS) to address the endogeneity issue. By using instruments which are exogenous to the dependent variable and highly correlated with our independent variable, we can “purge” the endogenous component of the independent variable, and assess whether its effect on our dependent variable still holds (Baum, Schaffer and Stillman 2007; Woolridge, 2012; Gujarati and Porter, 2012).

The IV-2SLS model needs to meet a few assumptions. First, the instruments must effectively predict the endogenous independent variable and independently account for a significant amount of the variance in the first-stage model. Second, the instruments and the error term of the second-stage model should be orthogonal—i.e., the excluded instruments should not independently predict the dependent variable in the second stage model once controlling for the other variables in the model. Third, the instrumented independent variable should  continue to exhibit a relationship with the dependent variable (Baum, Schaffer and Stillman 2007; Woolridge, 2012; Gujarati and Porter, 2012).

Our search for variables that meet the tests for appropriate instrumental variables presents a difficult challenge using data from the CANES. This is a general data set that does not include a wide range of possible instruments for immigration attitudes, and possible instrumental variables are available in some years but not in others. Fortunately, the 2016 American National Election Survey (ANES) includes a wide range of potential instruments. Following the general rules of the IV-2SLS approach, we have identified three variables as exogenous (or excluded) instruments that have no prior theoretical connections with welfare attitudes: (1) feelings when seeing the American flag flying; (2) opinion on whether government wiretap powers has gone too far; and (3) support for government encouragement of outsourcing. Although certain methodologists argue that most instrumental variables potentially violate the exclusion-restriction assumption (Mellon 2021) and researchers should be cautious when using behavior to estimate behavior, numerous published research articles used opinion-based instrumental variables to test endogeneity. For example, in one of the most important works that establishes the connection between immigration and welfare attitudes, Garand et al.’s 2017 AJPS article used three attitudinal variables as instrumental variables to tackle the immigration-welfare reverse causality issue.^[[2]](#endnote-2)^  Other scholars have adopted a similar approach and also used attitudinal variables as instrumental variables (Best and Krueger 2011; Hutchison 2014; Essig et al 2020)

Following these examples, we use these three instruments to estimate a two-stage model. In the first stage, we estimate a model that depicts our pro-immigration scale as a function of the three instrumental variables as well as the various control variables from our original models. The predicted values represent an instrumented measure of pro-immigration attitudes that is purged of the influence of welfare attitudes. We then use the purged (or instrumented) immigrant attitude variable to predict our dependent variable, support for welfare spending. In Appendix 3 Table 1 Model (2) we display the results for the second-stage model, and in Appendix 3 Table 1 Model (1) we present the results for the first-stage model.

We have conducted two diagnostic tests to determine whether or not our instruments are valid.  First, we use the Stock-Yogo weak identification test to determine whether or not these three instruments can sufficiently predict the endogenous independent variable, our pro-immigration scale (Stock and Yogo 2005). The Cragg-Donald F statistic we have obtained is 35.16, which exceeds the Stock-Yogo weak ID test critical value 9.08 (Stock and Yogo 2005; Baum Schaffer and Stillman 2007). Therefore we can reject the null hypothesis that our instruments are weak.

In the second test, we assess the possibility that our excluded instruments can predict the error terms from our second-stage models and thus suggest that they independently relate to the dependent variables; the Sargan statistic serves this purpose (Sargan 1988; Baum, Schaffer and Stillman 2003; Baum, Schaffer and Stillman 2007). The Sargan statistic for our model is 1.746 (p = 0.4178), which does not approach conventional levels of statistical significance (p < 0.05). As a result, we have little confidence that the excluded instruments could predict the second-stage models’ error terms.  In other words, the excluded instruments can be safely left out of the second stage models, as they would not independently predict welfare spending attitudes or attitudes toward welfare recipients.

Finally, and most importantly, our results in Appendix 3 Table 1 Model (2) shows that the purged (or instrumented) pro-immigration scale still has the expected positive effect on the dependent variable. In Model (1), the coefficient for the instrumented pro-immigration scale (b = 0.84, p = 0.000) is positive and far surpasses conventional levels of statistical significance, indicating that immigration attitudes have a strong effect on attitudes toward welfare recipients even when we control for endogeneity. After accounting for possible endogeneity, we still find that individuals who hold more favorable immigration attitudes remain significantly and strongly more likely to have higher levels of support for welfare spending.

Taken together, these findings show strong support for a causal arrow that leads from immigration attitudes to welfare attitudes. Our immigration attitudes variables have a strong effect on welfare attitudes even after we consider possible endogeneity. The 2016 results give us confidence that immigration attitudes should have a similar effect on welfare attitudes in our analyses of CANES data. These findings correspond with results from experimental research by Alesina et al. (2023) who conclude “the direction of causality is from perceptions of immigrants to support for redistribution rather than the other way around” (29).

**Appendix Table 4.1. First-stage and second-stage models for the IV-2SLS analyses, 2016 American National Election Study**

1. To generate the percentage of Black households on welfare, we employ a similar procedure to that used to measure immigrant welfare use. [↑](#endnote-ref-1)
2. In Garand et al.’s 2017 AJPS paper, they used the following three attitudinal variables as instrumental variables: (1) support for the requirement that the government be required to obtain a warrant to authorize wiretapping of phone calls by American citizens who are suspected of being terrorists; (2) support for limits on foreign imports; and (3) degree of agreement with the assertion that immigrants take away jobs from “people already here.” With these three instrumental variables, they conducted IV‐2SLS analyses to show that “how Americans think of immigration continues to have a strong, independent effect on their attitudes toward welfare recipients and their support for welfare spending in the United States even after we account for endogeneity” (Garand et al. 2017; Supporting Information Page 7).   [↑](#endnote-ref-2)
